# Carboplatin-induced gene expression changes *in vitro* are prognostic of survival in epithelial ovarian cancer

**DOI:** 10.1186/1755-8794-1-59

**Published:** 2008-11-28

**Authors:** Panagiotis A Konstantinopoulos, Elena Fountzilas, Kamana Pillay, Luiz F Zerbini, Towia A Libermann, Stephen A Cannistra, Dimitrios Spentzos

**Affiliations:** 1 Division of Hematology/Oncology, Beth Israel Deaconess Medical Center, Harvard Medical School, Boston, Massachusetts, USA; 2 Division of Interdisciplinary Medicine and Biotechnology, Beth Israel Deaconess Medical Center, Harvard Medical School, Boston, Massachusetts, USA

## Abstract

**Background:**

We performed a time-course microarray experiment to define the transcriptional response to carboplatin *in vitro*, and to correlate this with clinical outcome in epithelial ovarian cancer (EOC). RNA was isolated from carboplatin and control-treated 36M2 ovarian cancer cells at several time points, followed by oligonucleotide microarray hybridization. Carboplatin induced changes in gene expression were assessed at the single gene as well as at the pathway level. Clinical validation was performed in publicly available microarray datasets using disease free and overall survival endpoints.

**Results:**

Time-course and pathway analyses identified 317 genes and 40 pathways (designated time-course and pathway signatures) deregulated following carboplatin exposure. Both types of signatures were validated in two separate platinum-treated ovarian and NSCLC cell lines using published microarray data. Expression of time-course and pathway signature genes distinguished between patients with unfavorable and favorable survival in two independent ovarian cancer datasets. Among the pathways most highly induced by carboplatin *in vitro*, the NRF2, NF-kB, and cytokine and inflammatory response pathways were also found to be upregulated prior to chemotherapy exposure in poor prognosis tumors.

**Conclusion:**

Dynamic assessment of gene expression following carboplatin exposure *in vitro *can identify both genes and pathways that are correlated with clinical outcome. The functional relevance of this observation for better understanding the mechanisms of drug resistance in EOC will require further evaluation.

## Background

Epithelial ovarian cancer (EOC) is the leading cause of cancer mortality from gynecologic malignancies [1]. The majority of patients present with advanced disease that is typically managed with surgical cytoreduction followed by postoperative chemotherapy [2]. Platinum analogs including carboplatin (CBDCA, cis-diammine-1,1-cyclobutane dicarboxylate platinum) are the mainstay of treatment yielding response rates of approximately 70% in newly diagnosed patients with advanced disease [3,4]. However, the major limitation to the successful treatment of EOC is the frequent development of disease recurrence, followed by resistance to chemotherapy, resulting in an overall 5-year survival of only 20–30% for patients with advanced disease.

In an attempt to understand the mechanisms of chemotherapy resistance, several studies have used microarray technology to assess tumor gene expression at the time of diagnosis or at the time of subsequent recurrence [5-8]. A limitation of this approach is the difficulty of obtaining tumor tissue at the time of relapse, and the fact that such tissue may not be fully representative of the mechanisms that mediate drug response early in the patient course, at a time when first line chemotherapy is being administered.

In order to overcome these limitations, we wished to determine whether a dynamic assessment of gene expression after carboplatin exposure *in vitro *might have clinical relevance and provide insights into the mechanisms of chemoresistance. For this purpose, we employed time-course and pathway analysis approaches in order to capture changes in individual genes as well as gene pathways [9-12]. The significance of using this approach for pathway discovery in EOC is discussed.

## Methods

### Cell line

The 36M2 human EOC cell line was developed in our laboratory as previously reported [13-15]. This cell line was derived from serial passage of ovarian serous carcinoma cells in nude mice and exhibits clinical and histologic characteristics similar to papillary serous EOC in humans. It was grown in RPMI 1640 media with L-glutamine, 10% fetal bovine serum and 1% of penicillin/streptomycin.

### Cell death detection assay

Cell death was measured using the Cell Death Detection ELISA (Roche Diagnostics, GmBH, Mannheim Germany), according to manufacturer's instructions. Cell Death Detection ELISA is a photometric enzyme immunoassay that allows qualitative and quantitative *in vitro *determination of cytoplasmic histone-associated fragments (mono- and oligonucleosomes). In brief, after cell lysis and centrifugation, the cytoplasmic fractions were prediluted 1:10 with incubation buffer that contained both biotin-conjugated mouse monoclonal anti-histone antibodies and horseradish peroxidase-conjugated mouse monoclonal anti-DNA antibodies (sandwich ELISA principle). Quantification of DNA fragments (a surrogate of *in vitro *apoptosis) was done by photometric determination of horseradish peroxidase using ABTS (2,2'-azino-di [3-ethylbenzthiazoline sulfonate]) [16].

### Design of time-course microarray experiment

We treated the 36M2 cell line with varying concentrations (0.1 μM to 200 μM) of carboplatin for 24 hrs. Cells were then washed thoroughly with PBS and allowed to grow in fresh medium. Cell death was assessed for each carboplatin dosage at distinct time points using the previously described ELISA assay.

Doses of carboplatin up to 10 μM resulted in apoptotic cell death similar to control at all time points, while treatment with 200 μM was associated with significant apoptotic death at early time points (see Additional File 1). Treatment with 100 μM resulted in early apoptotic changes at 36 hours, followed by significant cell death at 48 hours (see Additional File 2).

Since our goal was to capture the transcriptional response to carboplatin up until early apoptotic changes became evident, we treated the 36M2 cell line with carboplatin 100 μM or vehicle-control for 24 hrs and cells were harvested and processed for RNA isolation at 24, 30 and 36 hrs after treatment. We did not study time points beyond 36 hrs since significant apoptotic cell death could obscure the transcriptional response to carboplatin. All data for all time points were obtained in duplicates.

### RNA Isolation and Affymetrix GeneChip Hybridization

Total RNA was isolated using the RNeasy Mini Kit (Qiagen Valencia, CA, USA) according to manufacturer's instructions. cDNA synthesis and hybridization on oligonucleotide microarrays (U133 Plus 2.0 Array GeneChip, Affymetrix, Inc., Santa Clara, CA) containing approximately 54,700 transcripts were carried out using standard protocols. Microarray experiments were performed at the Beth Israel Deaconess Medical Center (BIDMC) Genomics Core [8,17,18] as previously described. Raw data were processed using Robust Multi-Array (RMA) analysis [19]. Signal intensities were normalized, background corrected, and bottom-trimmed at signal intensity of 50. Genes were filtered out if their log intensity variation percentile was less than 25% and/or if they were absent in more than 85% of the experiments. All raw microarray data are provided in GEO, accession number: GSE13525.

### Statistical Methods

#### a. Time series analysis

Time series analysis was performed using BRB-ArrayTools Version 3.6 [developed by Dr Richard Simon (Biometrics Research Branch, National Cancer Institute, Bethesda, MD)]. Time series analysis is a regression analysis of time course microarray data (see Additional File 3). This approach applies a regression model to estimate the effect of the interaction between time and class (in this case class reflects treated versus control cells) and identifies genes whose variation of expression over time was different between carboplatin and control treated cells. Statistical significance for this model was set at 0.01 at a false discovery rate of 0.15.

#### b. Pathway Analysis

Pathway analysis was performed using the gene set comparison tool of BRB-ArrayTools Version 3.6 (Biometrics Research Branch, National Cancer Institute). We analyzed all predefined Biocarta pathways (obtained through the NCI public database) for differential expression between the carboplatin-treated and the vehicle-treated cells. Biocarta' is a trademark of Biocarta Inc. (San Diego, CA).

The statistical significance for differential expression of each pathway was estimated using the functional class scoring method [20]. In brief, a p value was computed for each gene in each pathway and then the set of p-values for each pathway was summarized by the LS score (mean negative natural logarithm of the p-values of the respective single gene univariate test) and the Kolmogorov-Smirnov (KS) score [20]. For each pathway, significance was assessed by testing the null hypothesis that the list of differentially expressed genes from each pathway was a random selection from the entire project gene list. N genes (equal to the number of genes of the pathway) were randomly selected from the project gene list, and the LS and KS statistics and their random distribution were computed (100,000 random selections). The LS (KS) permutation p-value was defined as the proportion of random simulations for which the LS (KS) statistic was larger than the LS (KS) statistic computed for the pathway with the original gene list. Statistical significance was set at 0.005.

#### c. Hierarchical Clustering

Publicly available gene expression data from the ovarian cancer cell line A2780 and the non-small cell lung cancer (NSCLC) cell line A549 were used for unsupervised hierarchical clustering with the average linkage method as implemented in the BRB Array Tools Version 3.6. All genes were median-centered across the experiments.

The gene signatures used for hierarchical clustering were mapped across different platforms (from U133 Plus 2.0 to U95Av2 platforms and from U133 Plus 2.0 to U133A platforms) using the Affymetrix 'best match' tool. Mapped time-course and pathway signatures were used without additional filtering for unsupervised hierarchical clustering.

#### d. Survival Analysis

In order to evaluate whether the gene expression signatures associated with carboplatin exposure provided clinically relevant information, we used two independent, clinically annotated, publicly available microarray datasets previously published by our group and others ( and , respectively) [21,22]. In regard to our dataset, the study protocol for collection of tissue and clinical information was approved by the institutional review board at our institution and patients provided written informed consent authorizing the collection and use of the tissue for study purposes.

We tested whether these signatures would predict disease free survival (DFS) and overall survival (OS) using the Survival Risk Prediction Algorithm as implemented in BRB-ArrayTools Version 3.6. We used the DFS and OS definitions exactly as used in these previously published studies. Gene signatures were mapped from U133 Plus 2.0 to U95Av2 platforms and from U133 Plus 2.0 to U133A platforms using the Affymetrix 'best match' tool.

The methodological principles of the Survival Risk Prediction Algorithm have been previously described [23]. In brief, a high and a low-risk survival groups were defined based on a multivariate model of the expression level of the genes contained in each gene signature and the Cox regression coefficient for each gene (supervised principal component method). This multivariate model was used in a leave-one-out cross validation process to assign risk-group membership for clinical samples. Kaplan-Meier DFS and OS curves were plotted for two risk groups, with higher or lower than median risk of death or recurrence. Statistical significance of the survival splits was assessed by the log-rank test and a permutation statistic was calculated by randomly reassigning the survival data among cases, repeating the entire survival risk prediction 100 times and estimating how many times the log-rank statistic is lower than the log-rank statistic for the real data. This represented the permutation significance level for testing the null hypothesis that there is no relation between the expression data and survival.

## Results

### Time-course analysis of gene expression after carboplatin exposure

36M2 cells were treated with carboplatin 100 μM or vehicle-control for 24 hrs, and RNA was isolated in duplicate at selected time points (as described in Material and Methods) from initiation of exposure until early apoptotic changes became apparent. Using time-series analysis to assess the interaction between time and class (carboplatin versus vehicle-treated cells), we identified 317 genes that exhibited differential, time-dependent expression between carboplatin and vehicle treated cells. These 317 genes represented several different biological processes (as defined by Gene Ontology, Table 1) including "response to external stimulus," "surface receptor signal transduction," "cell proliferation," "cell-cycle regulation," and "cell adhesion." The 10 most upregulated genes after carboplatin exposure are shown in Table 2. In order to support the validation of these carboplatin related genes we investigated the expression of the 10 most upregulated genes identified in our analysis in another dataset that included gene expression data (Affymetrix U95Av2 platform) from A2780 ovarian cancer cells treated either with cisplatin or control [24]. Despite the different experiment design, and the different cell line, and after mapping probesets from U133 to U95, 7 genes (GDF15, GADD45A, ATF3, IL8, IL6, MAFF and TNFAIP3), were also upregulated in cisplatin treated cells versus control.

**Table 1:** Selected biological processes represented in the time-course signature.

Gene Ontology Biological Processes	Genes
1 Response to external stimulus	6
2 Cell-cell signaling	7
3 Cell surface receptor linked signal transduction	11
4 Cell proliferation	10
5 Defense response	10
6 Cell cycle arrest	6
7 Regulation of cell proliferation	6
8 Signal transduction	20
9 Negative regulation of progression through cell cycle	7
10 Cell adhesion	6

**Table 2:** The 10 most upregulated genes after at 36 hours after carboplatin exposure compared to baseline.

GENE NAME	GENE SYMBOL	FOLD CHANGE
Activating transcription factor 3	ATF3	17.2
Interleukin 8	IL8	13.4
Interleukin 6 (interferon, beta 2)	IL6	8.5
Growth differentiation factor 15	GDF15	7
Chromosome 1 open reading frame 79	C1orf79	6.3
Pentraxin-related gene, rapidly induced by IL-1 beta	PTX3	6.3
v-maf musculoaponeurotic fibrosarcoma oncogene homolog F (avian)	MAFF	6
Claudin 1	CLDN1	5.9
Growth arrest and DNA-damage-inducible, alpha	GADD45A	5.7
Tumor necrosis factor, alpha-induced protein 3	TNFAIP3	4.9

**Figure 1:**
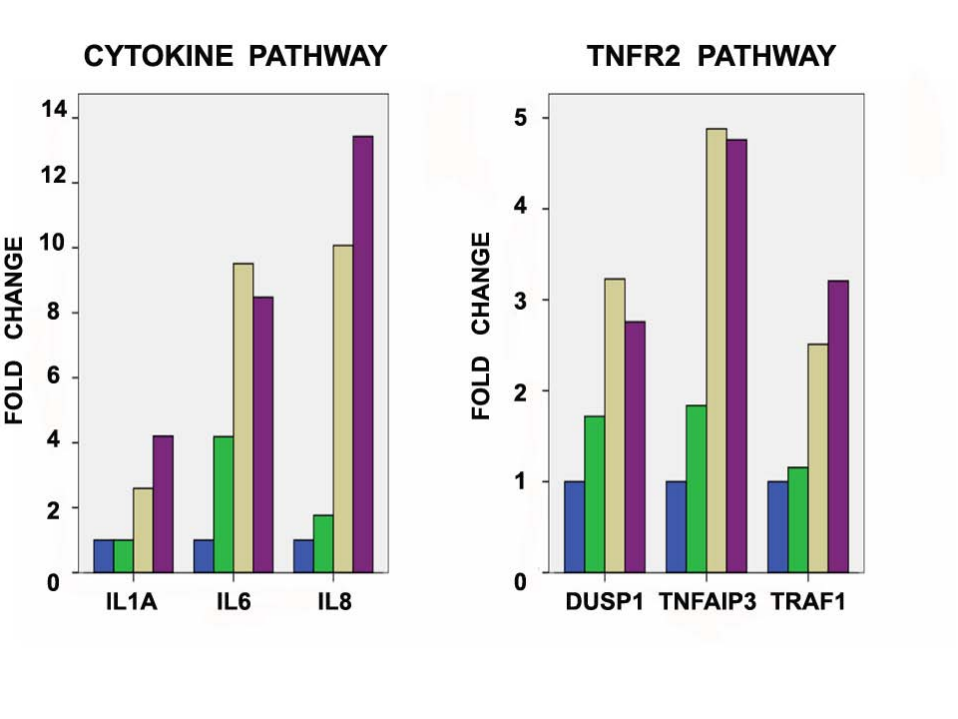
**Over time fold expression changes (compared to baseline-0 hours) of selected genes in the TNFR2 and cytokine pathways in carboplatin-treated 36 M2 cells**. Only treated cells are included and fold change values are in reference to baseline-0 hours. Blue, green, brown, and violet bars denote fold expression changes at 0, 24, 30, 36 hours, respectively. All changes shown are statistically significant (p < 0.001, F-test for comparison between time points).

Several of these genes belong to previously described signaling pathways, namely the TNFR2 signaling pathway (TNFAIP3), the ATM signaling pathway (GADD45A), the NF-kB signaling pathway (TNFAIP3), the cytokine and inflammatory response pathway (IL6 and IL8) and the oxidative stress response via NRF2 pathway (MAFF). As an example of the expression changes induced by carboplatin exposure, Figure 1 illustrates the over time fold expression changes of selected genes of two of these pathways (TNFR2 and cytokine and inflammatory response pathways). A complete list of all 317 genes (designated as "time-course signature") is included in Additional File 4.

**Figure 2:**
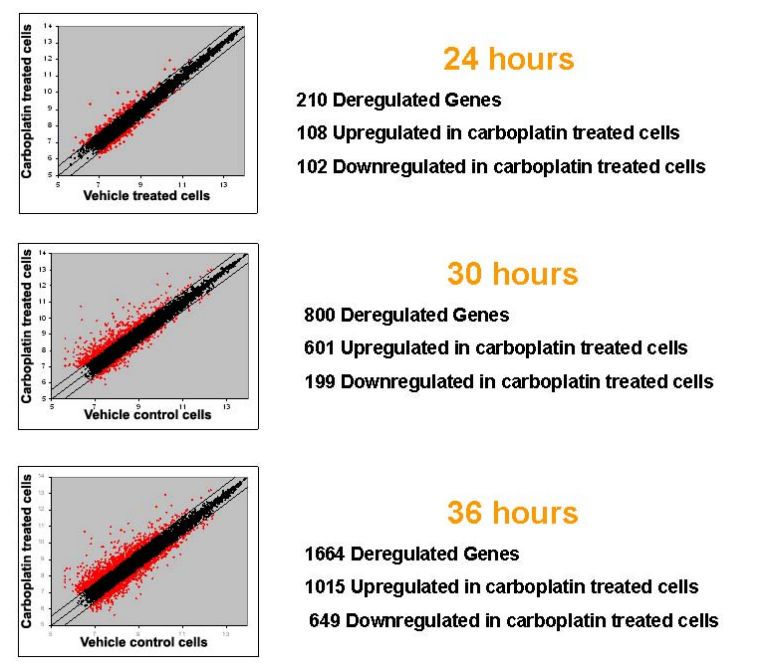
**Scatterplots showing differentially-expressed genes between carboplatin and vehicle-treated cells at 24, 30 and 36 hours**. Outlier lines show 1.5 fold differences between carboplatin and vehicle-treated cells.

Furthermore, we identified the deregulated genes between carboplatin and vehicle-treated cells for each time point (Figure 2). Specifically 24, 30 and 36 hours after carboplatin exposure 210, 800 and 1664 genes (see Additional File 5) were differentially expressed between carboplatin and vehicle-treated cells respectively (108 out of 210, 601 out of 800 and 1015 out of 1664 were upregulated in carboplatin treated cells). Of note 88% of the genes (281 out of 317 genes) in the time course signature were among the genes deregulated between treatment and control.

### Dynamic assessment of signaling pathways induced by carboplatin exposure

We hypothesized that individual genes that are differentially regulated after carboplatin exposure (Table 2) might be surrogates for carboplatin-induced changes in their corresponding pathways. Conversely, we considered the possibility that pathways may also be differentially regulated following carboplatin exposure, which might not be captured by the behavior of individual genes. Accordingly, we performed pathway analysis as previously described [20] and identified a total of 40 pathways that were differentially expressed between carboplatin-treated and vehicle-treated cells at a significance level of *p *= 0.005. This 40 pathway list (Table 3) includes diverse signaling pathways associated with apoptosis, response to DNA damage, cytokine signaling, DNA repair, mitogenesis and antioxidant response. The 270 genes comprising all 40 signaling pathways (designated as 'pathway signature') are provided in Additional File 6.

**Table 3:** Pathways deregulated after carboplatin exposure.

	**Pathway description**	**Genes**	P-Value *
1	Oxidative Stress Induced Gene Expression Via Nrf2	29	< 0.0001
2	ATM Signaling Pathway	23	< 0.0001
3	Role of EGF Receptor Transactivation by GPCRs in Cardiac Hypertrophy	16	< 0.0001
4	Pertussis toxin-insensitive CCR5 Signaling in Macrophage	12	< 0.0001
5	Cadmium induces DNA synthesis and proliferation in macrophages	14	< 0.0001
6	Cytokine Network	5	< 0.0001
7	Adhesion and Diapedesis of Granulocytes	6	< 0.0001
8	IL12 and Stat4 Dependent Signaling Pathway in Th1 Development	7	< 0.0001
9	Signal transduction through IL1R	27	< 0.0001
10	Cells and Molecules involved in local acute inflammatory response	12	< 0.0001
11	Adhesion and Diapedesis of Lymphocytes	10	< 0.0001
12	Bone Remodelling	12	< 0.0001
13	Regulation of hematopoiesis by cytokines	5	< 0.0001
14	Cytokines and Inflammatory Response	9	< 0.0001
15	IL 17 Signaling Pathway	8	< 0.0001
16	METS affect on Macrophage Differentiation	20	< 0.0001
17	NF-kB Signaling Pathway	22	< 0.0001
18	The 4-1BB-dependent immune response	16	0.000124
19	Inhibition of Cellular Proliferation by Gleevec	24	0.000125
20	Mechanism of Gene Regulation by Peroxisome Proliferators via PPARa	47	0.000132
21	TNFR2 Signaling Pathway	16	0.00031
22	Activation of PKC through G protein coupled receptor	7	0.000423
23	TPO Signaling Pathway	30	0.000472
24	Eukaryotic protein translation	17	0.000796
25	Erythropoietin mediated neuroprotection through NF-kB	9	0.001252
26	NFkB activation by Nontypeable Hemophilus influenzae	29	0.001499
27	IFN alpha signaling pathway	12	0.00188
28	The information-processing pathway at the IFN-beta enhancer	15	0.002252
29	Hypoxia-Inducible Factor in the Cardiovascular System	22	0.002535
30	Free Radical Induced Apoptosis	10	0.002556
31	FAS signaling pathway (CD95)	45	0.002569
32	Keratinocyte Differentiation	39	0.002711
33	Neuropeptides VIP and PACAP inhibit the apoptosis of activated T cells	14	0.00285
34	Repression of Pain Sensation by the Transcriptional Regulator DREAM	13	0.002997
35	CD40L Signaling Pathway	17	0.003313
36	Nitric Oxide Signaling Pathway	6	0.0017
37	Induction of apoptosis through DR3 and DR4/5 Death Receptors	34	0.0046
38	SREBP control of lipid synthesis	7	0.0049
39	Regulation of transcriptional activity by PML	18	0.0029843
40	Double Stranded RNA Induced Gene Expression	10	0.0015

* By Functional Class Scoring Method

### External validation of time course and pathway signatures

In order to demonstrate the reproducibility of the time-course and pathway signatures we utilized two previously published microarray datasets from different laboratories. The first dataset included gene expression data (Affymetrix U95Av2 platform) from A2780 ovarian cancer cells [24] treated either with cisplatin or control. Despite the change in microarray platform, both time-course and pathway signatures successfully distinguished between cisplatin and vehicle-treated A2780 cells (see Additional File 7).

The second dataset included gene expression data (Affymetrix U133A platform) from the A549 NSCLC cell line that was treated for 24 hr with vehicle control, or carboplatin [25]. Again, both signatures successfully separated the carboplatin- and vehicle-treated A549 samples (see Additional File 8) indicating that the time-course and pathway signatures are reproducible, even in a non-ovarian cancer cell line.

### Clinical relevance of time course and pathway signatures

We have shown that exposure to carboplatin *in vitro *induced a characteristic change in the expression of several genes and their corresponding pathways. In order to determine whether this observation has clinical relevance, we investigated whether the time course or pathway analysis correlated with disease free survival (DFS) and overall survival (OS) using two separate, clinically annotated ovarian cancer microarray datasets. The first dataset (Dataset 1, run on Affymetrix U95Av2 arrays, previously published work by our group [8]) included 66 ovarian cancers and the second dataset (Dataset 2, run on Affymetrix U133A arrays) included 133 ovarian cancers [21]. DFS and OS data were available for Dataset 1, while only OS data were available for Dataset 2.

**Figure 3:**
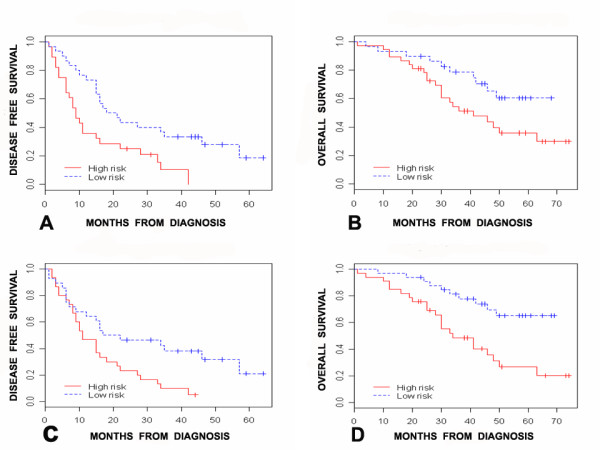
**Association between *in vitro *signatures and clinical outcome (Dataset 1)**. (A): Association between time course signature and DFS: Median DFS: 9 versus 18 months for the unfavorable and favorable groups respectively (p = 0.005, log-rank test), Hazard Ratio 2.3 (95% C.I. 1.3 – 4.2). (B): Association between time course signature and OS: Median OS: 41 months versus not yet reached for the unfavorable versus favorable group (p = 0.043, log-rank test), Hazard Ratio 2.1 (95% C.I. 1 – 4.5). (C): Association between pathway signature and DFS: Median DFS: 11 versus 17 months for the unfavorable versus favorable group, (p = 0.011, log-rank test), Hazard Ratio 2.1 (95% C.I. 1.2 – 4). (D): Association between pathway signature and OS: Median OS: 34 months versus not yet reached for the unfavorable versus favorable group (p = 0.002, log-rank test), Hazard Ratio 3.1 (95% C.I. 1.5 – 6.6).

We used the U95Av2-mapped subset of the time-course signature (obtained through the Affymetrix 'best match' algorithm) for survival risk prediction on Clinical Dataset 1. Despite the change in platform, survival risk prediction using the U95Av2-mapped subset of the time-course signature distinguished between a high and a low-risk group for DFS (median 9 versus 18 months, log-rank p = 0.005, permutation p = 0.04, Figure 3A) and OS (median 41 months versus not yet reached, log rank p = 0.04, permutation p = 0.11, Figure 3B). Similarly, the U133A-mapped subset of the time-course signature discriminated between a low-risk and a high-risk group in Dataset 2 (median OS 33 versus 118 months, log-rank p = 0.001, permutation p = 0.02, see Additional File 9, panel A).

As with the time-course signature, survival risk prediction using the U95A2-mapped subset of the pathway signature distinguished between patients with unfavorable and favorable DFS in Dataset 1, (median DFS 11 versus 17 months, log-rank p = 0.01, permutation p = 0.08, Figure 3C), and OS (median OS 34 months versus not yet reached, log-rank p = 0.002, permutation p = 0.05, Figure 3D). Similarly, the U133A-mapped subset of the pathway signature distinguished between patients with unfavorable and favorable OS in Dataset 2 (median OS 31 vs 112 months, log-rank p < 0.001, permutation p = 0.04, see Additional File 9, panel B).

Importantly, both pathway and time-course signatures were independently associated with DFS and OS when tested in multivariate analysis that included known prognostic factors of ovarian cancer including age and debulking status. Specifically, multivariate analysis showed that pathway signature was associated with DFS (adjusted Hazard Ratio: 2.04, 95% C.I. 1.1–3.78) and OS (adjusted Hazard Ratio: 2.2, 95% C.I. 1.05–4.57), while time-course signature was associated with DFS (adjusted Hazard Ratio: 2.52, 95% C.I. 1.36–4.67) and OS (adjusted Hazard Ratio: 1.96, 95% C.I. 0.92–4.2). There were no publicly available data regarding age and debulking status for Dataset 2.

### NRF2, NF-kB, and cytokine network genes are upregulated at baseline in tumors from patients with unfavorable outcome

Given the observation that carboplatin induced changes in gene expression had prognostic significance when applied to baseline, pre-chemotherapy tumor samples, we sought to identify which gene networks were upregulated in patients with poor outcome compared to those with good outcome, hypothesizing that they might be relevant to the development of chemoresistance. We analyzed the baseline expression levels of the 10 most upregulated genes after carboplatin exposure shown in Table 1, and their 5 corresponding pathways (TNFR2, ATM, NF-kB, NRF2 and cytokine and inflammatory response pathways), in tumors from patients with favorable and unfavorable DFS and OS as identified by the time course signature. Four of these genes (PTX3, GADD45A, IL-6, IL-8 and MAFF) were statistically significantly upregulated in patients with unfavorable DFS compared to those with favorable DFS. Furthermore, pathway analysis showed that 3 of the 5 pathways ("oxidative stress response via NRF2", "NF-kB signaling," and "cytokine and inflammatory response" pathways) were differentially expressed between unfavorable and favorable prognosis tumors (Table 4). Importantly, the same genes of these 3 pathways that were upregulated in unfavorable outcome tumors (Table 4), had already been shown to be upregulated after carboplatin exposure (Table 1, Figure 1).

**Table 4:** Selected genes of the NRF2, cytokine response and NF-kB signaling pathways upregulated in patients with unfavorable DFS and OS

**PATHWAY**	PATHWAY P VALUE *	**GENE**	GENE P VALUE**
**Oxidative stress response via Nrf2**	< 0.0001	MAFF	< 0.0001
		NRF2 (NFE2L2)	< 0.0001
		CRYZ	< 0.001
		CREB1	0.015
**Cytokine and Inflammatory Response**	< 0.0001	IL-6	0.005
		IL-8	0.013
		IL-15	0.0005
**NF-kB signaling pathway**	0.0002	NF-kB1	< 0.0001
		IL1-R1	0.003
		CHUK	0.002
		RELA	0.005

* By Functional Class Scoring Method

** By t-test

Finally, since these genes were upregulated at baseline in poor outcome tumors we analyzed whether they contained prognostic information. The combined expression levels of these genes were not associated with OS or DFS in either datasets. This strengthens the value of our approach to use dynamic gene expression as a mean to capture prognostic information.

## Discussion

In this study, we used time-course and pathway analysis to obtain a dynamic assessment of gene expression after carboplatin exposure *in vitro*. Time-course analysis formally assesses the statistical interaction between time (as a continuous variable) and class (in this case carboplatin versus vehicle-treated cells), while pathway analysis combines measurements across multiple gene members of each pathway to identify networks deregulated after carboplatin exposure that are typically missed in the single gene approach. Our analyses identified two gene expression signatures – designated time-course and pathway signatures, respectively – that are induced after carboplatin exposure (Tables 1 and 3). In order to assess the clinical relevance of these signatures, we investigated whether the expression of their genes in tumor specimens obtained at the time of surgery (prior to chemotherapy administration) was associated with clinical outcome. Interestingly, despite the different microarray platforms used, baseline, pre-chemotherapy expression of time-course and pathway signature genes distinguished between a high- and low-risk group of patients with unfavorable and favorable OS and DFS respectively, in two previously published independent clinically annotated microarray datasets. Given the dynamic nature of these signatures, their prognostic value when assessed in a static, pre-chemotherapy tumor sample obtained at diagnosis was unanticipated.

In order to further investigate this observation, we compared the baseline, pre-chemotherapy expression of the 10 most upregulated genes (Table 2) and their corresponding pathways, between tumors from patients with favorable and unfavorable DFS. Interestingly, 4 of the 10 genes (MAFF, IL-6, IL-8 and PTX3) and 3 of 5 pathways (NRF2 oxidative stress response, NF-kB signaling and cytokine and inflammatory response pathways) were statistically significantly upregulated in patients with unfavorable DFS or OS compared to those with favorable DFS or OS. The finding that some of the genes and pathways that are upregulated after carboplatin exposure *in vitro*, are also upregulated at baseline in tumors from patients with unfavorable DFS, suggests that they may be involved in mediating chemoresistance.

While the role of the NF-kB signaling pathway in platinum resistance has been extensively studied [26], emerging data indicate that the NRF2 signaling pathway may also be involved in this process (Table 4). Electrophilic xenobiotics, such as platinum analogs, induce dissociation of the transcription factor NRF2 from its cytoplasmic inhibitor Keap1 [27]. NRF2 then translocates to the nucleus, where it heterodimerizes with members of the small MAF family of proteins (including MAFF, among the 10 most upregulated genes in Table 2) to activate the transcription of antioxidant, xenobiotic detoxification and drug efflux pump genes that confer cytoprotection against drugs [28,29]. Members of the cytokine and inflammatory response pathway have also been associated with chemotherapy resistance, albeit not yet with platinum resistance. Specifically, IL-6, (again among the most 10 upregulated genes in Table 2) is reportedly upregulated after paclitaxel exposure *in vitro *and has been associated with paclitaxel resistance by inducing multidrug resistance gene-1 transcription with subsequent P-glycoprotein overexpression [30,31].

Importantly, we performed in silico validation of our *in vitro *time-course and pathway signatures using published microarray data from a different ovarian cancer cell line and a NSCLC cell line exposed to platinum in different laboratories. Despite the differences in microarray platforms in both cases, our signatures successfully distinguished between carboplatin and vehicle-treated cells confirming their association with carboplatin exposure irrespective of cell line selection. Additionally, the observation that both signatures discriminated between carboplatin and vehicle-treated lung cancer cells suggests that the gene networks involved in the transcriptional response to carboplatin may be shared by cancer cells, regardless of tissue of origin.

Further mechanistic insights may also be derived from examining the full content of the time-course and pathway signatures. Several pathways mediating apoptosis, DNA damage response, cytokine signaling, DNA repair, and mitogenesis were involved in cellular transcriptional response to carboplatin. Some of these pathways are known to be deregulated after platinum exposure including the ATM, FAS and epidermal growth factor receptor (EGFR) pathways [32-34]. However, other pathways not previously linked to platinum exposure were also identified by our pathway analysis, for example, the death receptor DR3, DR4/5 apoptotic pathway and the IL1R, IL-17, and tumor necrosis factor (TNFR2) pathways among others.

It is revealing that some of the 40 pathways identified by pathway analysis were not captured by time-course analysis. For instance, while none of the gene members of the PKC-GPCR (G-protein coupled receptor) pathway were included in the time course signature, pathway analysis indicated that this pathway is nonetheless involved in the cellular response to carboplatin, consistent with previous studies demonstrating its activation after platinum exposure and potential association with platinum resistance *in vitro *[35-37]. Conversely, not all genes identified by time course analysis were captured by pathway analysis, for example two genes among those with the highest fold change in the time-course signature, ATF3 (activating transcription factor 3) and claudin 1 (Table 2). These findings indicate that complementary insights into chemoresponse might be gained by analyzing the results of microarray time course experiments not only from the perspective of individual genes, but by considering gene families as defined by pathway analysis. Further studies will be necessary to define the precise role of these genes and pathways in promoting chemoresistance or chemosensitivity, and to evaluate their potential as novel drug targets in patients with EOC.

## Conclusion

In conclusion, this work demonstrates for the first time that systematic dynamic assessment of gene expression changes following carboplatin exposure in vitro, using formal time course and pathway statistical approaches, can identify genes and pathways associated with clinical outcome in ovarian cancer. Pathways highly induced in vitro, such as the NRF2, NF-kB, and cytokine and inflammatory response pathway, were shown for the first time to be upregulated in poor prognosis tumors in a statistically robust manner. This study provides proof of principle that comprehensive transcriptional assessment following exposure to chemotherapeutic agents in vitro provides information that can be translated to the clinical setting, as well as potential useful mechanistic insights into chemoresistance.

## Competing interests

The authors declare that they have no competing interests.

## Authors' contributions

All authors participated in the design, performance of the study and interpretation of the results. All authors read and approved the final manuscript.

## Pre-publication history

The pre-publication history for this paper can be accessed here:



## Supplementary Material

Additional file 1 **Apoptotic curve of 36M2 cells with various carboplatin concentrations**. This figure shows the apoptotic curve of 36M2 cells treated with varying concentrations of carboplatin (0.1 μM to 200 μM) for 24 hours. Click here for file

Additional file 2 **Early apoptotic changes at 36 hours with 100 μM of carboplatin**. Treatment with 100 μM of carboplatin resulted in early apoptotic changes at 36 hours, followed by significant cell death at 48 hours. Click here for file

Additional file 3 **Theoretical principles of time series analysis**. The theoretical principles of time series analysis as described in BRB Array Tools software. Click here for file

Additional file 4 **Time course signature**. A complete list of all 317 genes designated as time-course signature. Click here for file

Additional file 5 **Up and dowregulated genes for each time point**. List of all genes that are up- or downregulated between treatment and control for each time point. Click here for file

Additional file 6 **Pathway Signature**. The full list of the 270 genes comprising all 40 signaling pathways designated as pathway signature. Click here for file

Additional file 7 **Unsupervised hierarchical clustering of A2780 ovarian cancer cells**. Unsupervised hierarchical clustering using average linkage method of A2780 ovarian cancer cells exposed to cisplatin or control. Time course (A, left) and pathway signatures (B, right) successfully separate cisplatin and vehicle-treated A2780 cells. Click here for file

Additional file 8 **Unsupervised hierarchical clustering of A549 NSCLC cells**. Unsupervised hierarchical clustering using average linkage method of A549 NSCLC cells exposed to carboplatin or vehicle control. Time course (A, left) and pathway signatures (B, right) successfully separate carboplatin and vehicle-treated A549 cells. Click here for file

Additional file 9 **Association of time course and pathway signatures with OS in Dataset 2**. Association of time course and pathway signatures with OS in Dataset 2. A) Association between time course signature and OS [(median OS for the unfavorable and favorable groups was 33 and 118 months respectively (p = 0.001, log-rank test), hazard-ratio 2.2 (95% C.I. 1.4–3.6)]. B) Association between pathway signature and OS [(median OS for the unfavorable and favorable groups was 31 and 112 months respectively (p < 0.001, log-rank test), hazard-ratio 2.6 (95% C.I. 1.6–4.2)]. Click here for file
