# Supplementary figures and images for: Carboplatin-induced gene expression changes in vitro are prognostic of survival in epithelial ovarian cancer
Source: BMC Med Genomics. 2008 Nov 28;1:59. doi: 10.1186/1755-8794-1-59 (PMC2613398; doi:10.1186/1755-8794-1-59)

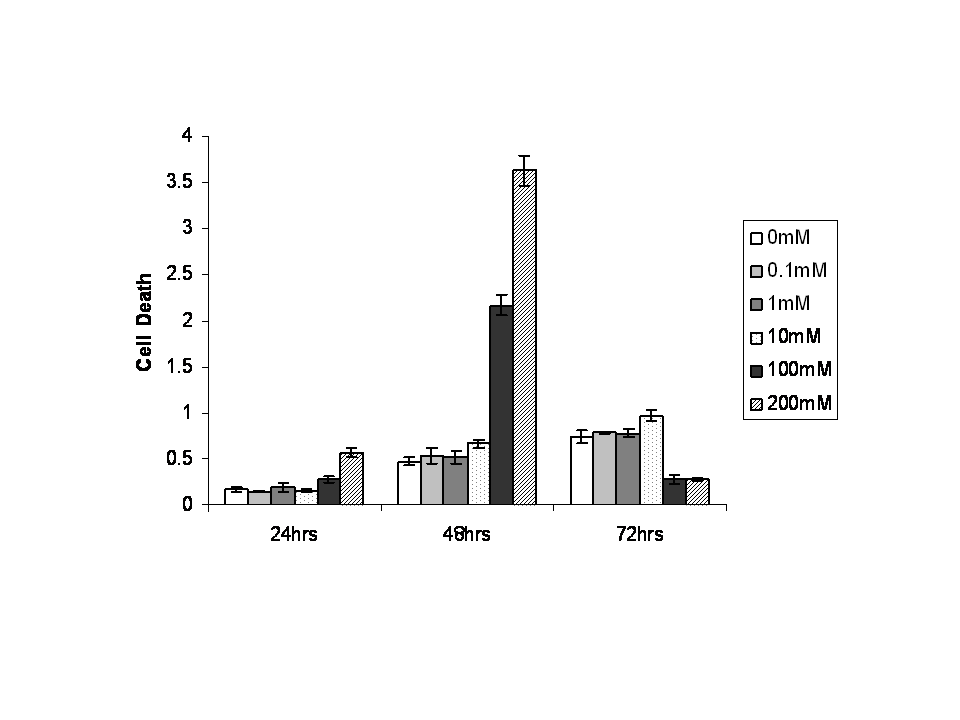

Supplement: Additional file 1 — Apoptotic curve of 36M2 cells with various carboplatin concentrations. This figure shows the apoptotic curve of 36M2 cells treated with varying concentrations of carboplatin (0.1 μM to 200 μM) for 24 hours. [file 1755-8794-1-59-S1.tiff]

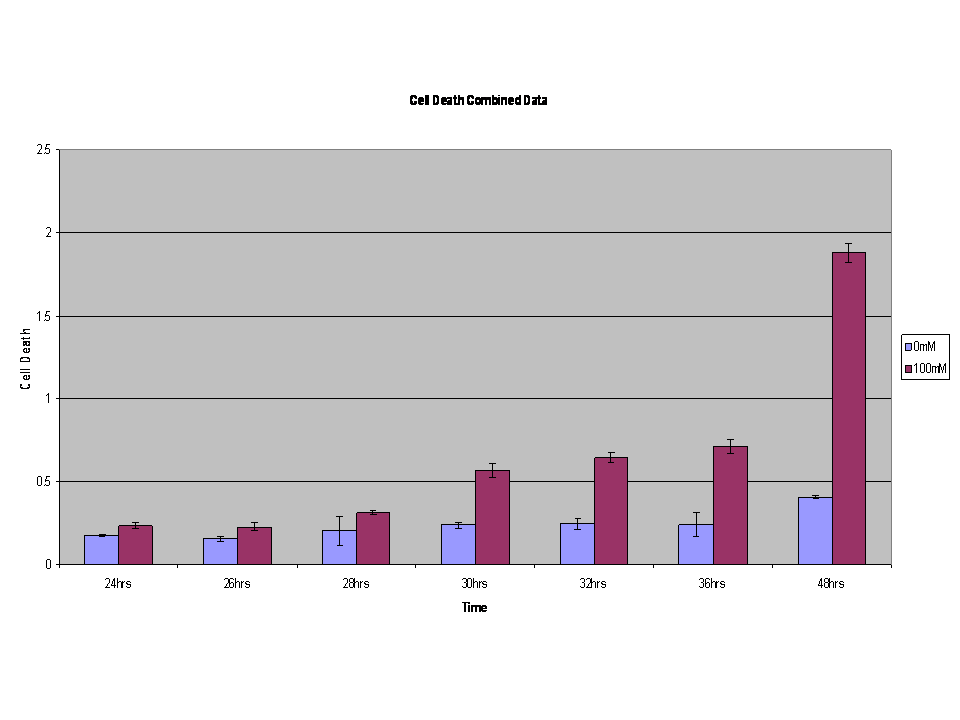

Supplement: Additional file 2 — Early apoptotic changes at 36 hours with 100 μM of carboplatin. Treatment with 100 μM of carboplatin resulted in early apoptotic changes at 36 hours, followed by significant cell death at 48 hours. [file 1755-8794-1-59-S2.tiff]

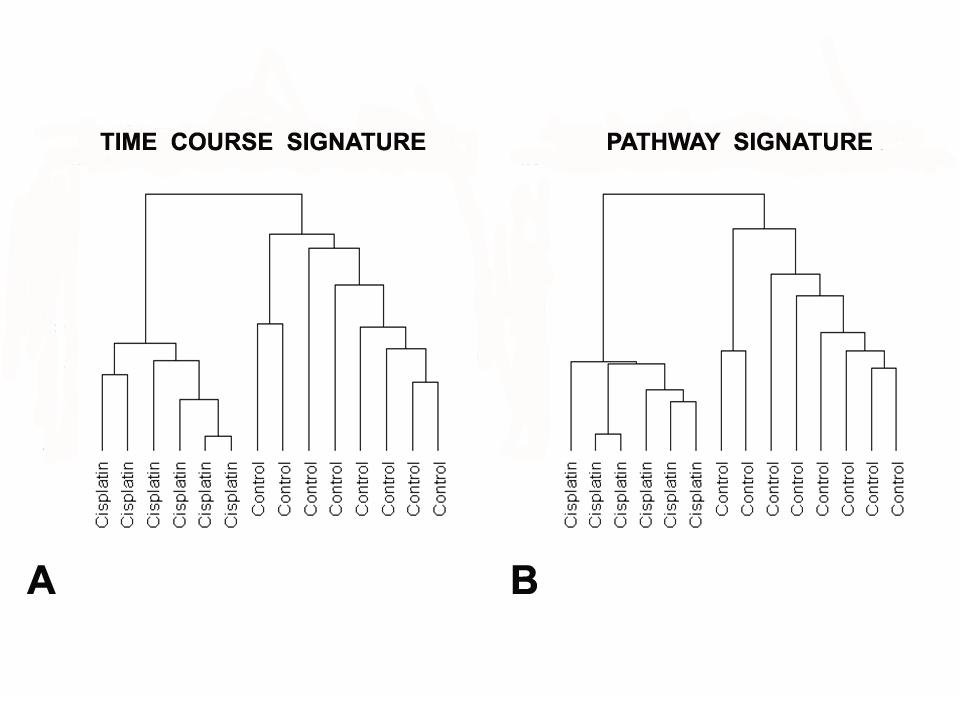

Supplement: Additional file 7 — Unsupervised hierarchical clustering of A2780 ovarian cancer cells. Unsupervised hierarchical clustering using average linkage method of A2780 ovarian cancer cells exposed to cisplatin or control. Time course (A, left) and pathway signatures (B, right) successfully separate cisplatin and vehicle-treated A2780 cells. [file 1755-8794-1-59-S7.tiff]

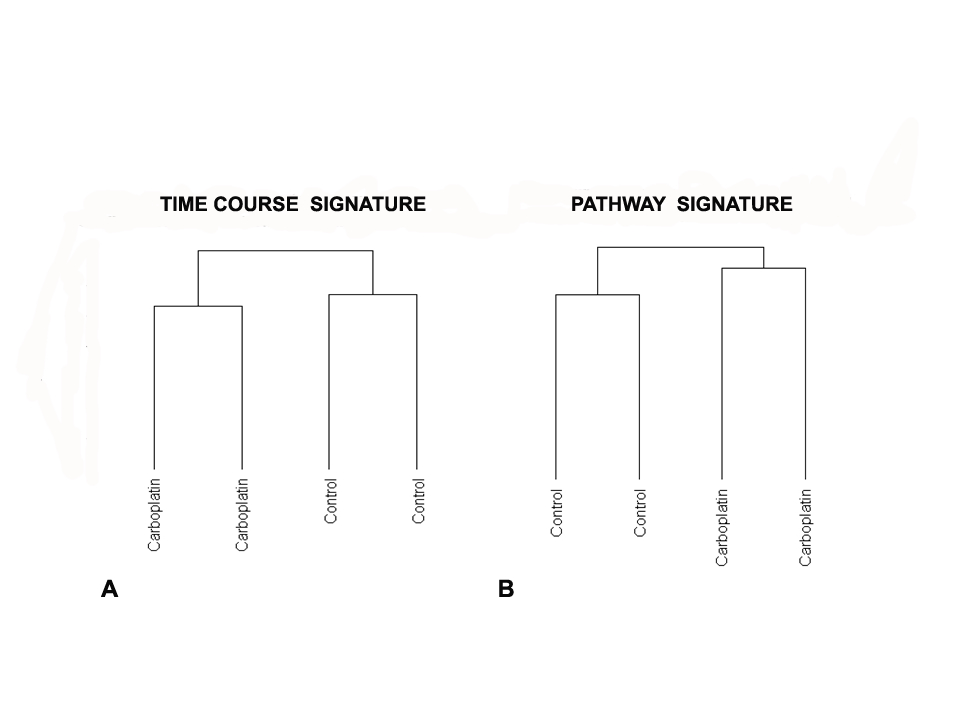

Supplement: Additional file 8 — Unsupervised hierarchical clustering of A549 NSCLC cells. Unsupervised hierarchical clustering using average linkage method of A549 NSCLC cells exposed to carboplatin or vehicle control. Time course (A, left) and pathway signatures (B, right) successfully separate carboplatin and vehicle-treated A549 cells. [file 1755-8794-1-59-S8.tiff]

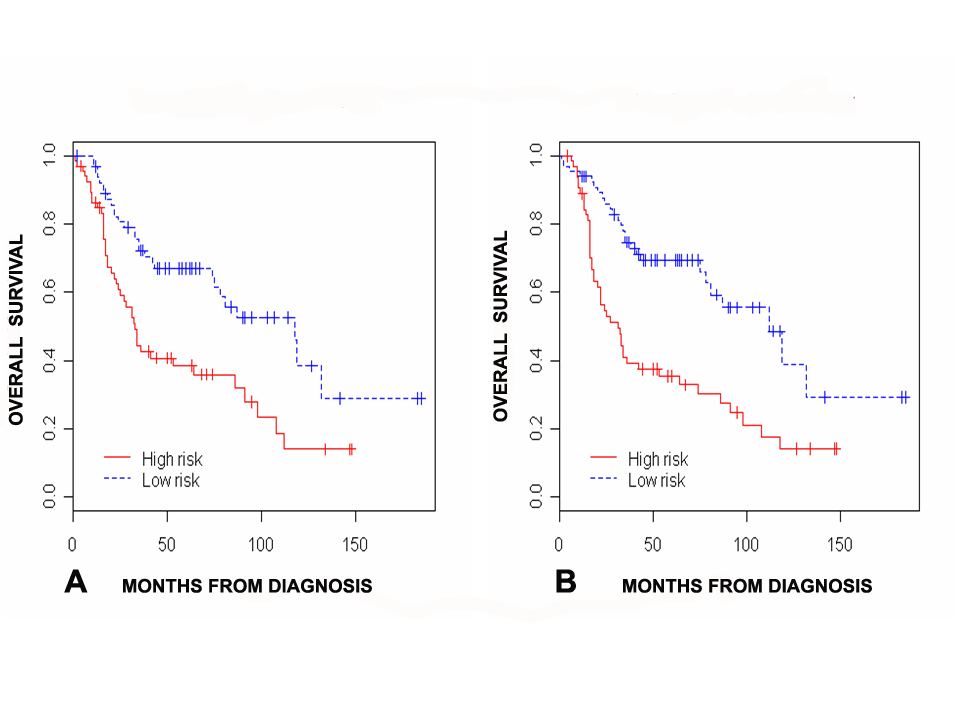

Supplement: Additional file 9 — Association of time course and pathway signatures with OS in Dataset 2. Association of time course and pathway signatures with OS in Dataset 2. A) Association between time course signature and OS [(median OS for the unfavorable and favorable groups was 33 and 118 months respectively (p = 0.001, log-rank test), hazard-ratio 2.2 (95% C.I. 1.4–3.6)]. B) Association between pathway signature and OS [(median OS for the unfavorable and favorable groups was 31 and 112 months respectively (p < 0.001, log-rank test), hazard-ratio 2.6 (95% C.I. 1.6–4.2)]. [file 1755-8794-1-59-S9.tiff]
