# Supplementary material for: Carboplatin-induced gene expression changes in vitro are prognostic of survival in epithelial ovarian cancer
Source: BMC Med Genomics. 2008 Nov 28;1:59. doi: 10.1186/1755-8794-1-59 (PMC2613398; doi:10.1186/1755-8794-1-59)
Supplement: Additional file 3 — Theoretical principles of time series analysis. The theoretical principles of time series analysis as described in BRB Array Tools software. [file 1755-8794-1-59-S3.doc]

********************************

** NAME OF THIS PLUG-IN: **

********************************

Time Series Analysis.

********************************

** PURPOSE OF THIS PLUG-IN: **

********************************

This plug-in can be used for regression analysis of time series

expression data. In its simplest form (model A), the genes whose

expression are varying over time are identified. A quadratic function

is fit to the expression data of each gene and the hypothesis is that the

linear and quadratic coefficients are simultaneously zero. The genes for

which this hypothesis is rejected are identified. The tests are performed

at a significance level specified by the user and also at a false discovery

rate (FDR) specified by the user. Two lists of significant genes are

produced, one for the specified significance level threshold and one for

the FDR threshold. To fit this model, the user must provide a column in

the experiment descriptor worksheet specifying the time point for each

array. This column should be strictly numeric and should not contain

alphabetic characters. The entry in the column should be blank if the

array is to be excluded from the analysis. The arrays at the same time

points can represent either technical or biological replicates, but

the two kinds of replicates should not be combined in the same analysis.

This plug-in is not appropriate for nested data where the same subject is

sampled at different time points.

Model B is for identifying genes that are changing over time, but

where there is a class variable to adjust for. For example, there could

be two strains of mice included in the experiment or arrays were from

two different print set batches. For model B it is assumed that the

variation in gene expression over time is the same for each class. The

output also indicates which genes are differentially expressed among

the classes uniformly over time.

Model C is similar to model B but the variation in gene expression over

time is permitted to differ among the classes. The output of model C

identifies these genes for which the variation over time is different

for different levels of the class variable. These genes are identified

based on the user specified significance level and based on the user

specified FDR. For genes whose variation over time does not significantly

vary among classes, model B is fit to determine whether the gene is varying

over time uniformly for each classes. Model C is useful for experiments

where the class variable represents a treatment indicator.

For data without a class variable, the ANOVA model takes the form:

y_{ijk} = alpha_i + beta_i t_j + lambda_i t_j**2 + e_{ijk},

e_{ijk} ~ NID(0,sigma**2) ......................................(A)

For data with a class variable, the ANOVA model takes the form

y_{ijkl} = alpha_i + beta_i t_j + lambda_i t_j**2 + delta_i x_l +

e_{ijkl},

e_{ijkl} ~ NID(0,sigma**2) ......................................(B)

and

y_{ijkl} = alpha_i + beta_i t_j + lambda_i t_j**2 + delta_i x_l +

gamma_i t_j x_l + rho_i t_j**2 x_l + e_{ijkl},

e_{ijkl} ~ NID(0,sigma**2) ......................................(C)

where

y_{ijk} or y_{ijkl} is the log ratio or log intensity,

alpha_i is the gene-specific average log intensity,

beta_i is the gene-specific time effect,

lambda_i is the gene-specific time**2 effect,

delta_i is the gene-specific class (variety) effect,

gamma_i is the gene-specific class*time interaction,

rho_i is the gene-specific class*time**2 interaction,

t_j is the time points,

x_l is the class variable,

e_{ikj} and e_{ikjl} are the random noise.

The F-test is based on the likelihood ratio test. Note that we assume

the random noise for each gene has the same standard deviation.

This assumption is used to increase the test power.

********************************

** USING THIS PLUG-IN: **

********************************

To run this function, the user should input the following:

. Column of exper descriptor sheet for time

. Column of exper descriptor sheet for class (optional)

. Column of exper descriptor sheet for indicator of included arrays

(optional)

. Threshold p value for testing effects

. Threshold false discovery rate (Benjamini & Hochberg, 1995) for testing effects

. OutputName

The elements of 'time' column should be numeric numbers.

For example '2.5 hours' is not allowed but '2.5' is OK.

The Column of exper descriptor sheet for class should be left empty if there is

only one level in this variable.

The "Column of exper descriptor sheet for indicator of included arrays"

is used to include arrays we are only interested in. For arrays we

don't want them to be included in analyses, we should leave blank value

in this column. We can put any value other than blank in this column

for arrays we are interested in. If nothing is specified in the dialog,

all arrays with non-empty factor labels will be used.

The result will be written to a html file in the folder

Project/Plugin/timeseries/Outputname where Outputname is specified in the dialog.

********************************

** OUTPUT OF THIS PLUG-IN: **

********************************

After finishing execution, an html file summarizing results will be opened

by internet explorer.

For data without 'class' variable, the ANOVA model (1) is fitted.

The numbers of significant genes by using the p-value/FDR criterion

are summarized in a hyper-linked html table.

For data containing 'class' variable, there are two hyper-linked summarized

tables. The first table shows the number of significant genes for testing

interaction term from model (2). For genes whose interaction is not significant,

we further fit them with model (3). The second table summarizes the number

of significant genes for 'time' and 'class' effects by using the p-value/FDR

criterion.

Rows of the gene list table are ordered by the p-value (or FDR) from the

smallest to the largest. Columns of the table includes gene identifiers,

p-value (or FDR) and coefficient estimates of the time effects

(Intercept=alpha_i, Time=beta_i, Time2=lambda_i).

The gene list table features drill-down linkage to NCBI databases

using clone, GenBank, or UniGene identifiers, and drill-down linkage

to the NetAffx database using Probeset ids.

The gene lists based on p-value or FDR criterion will be output to the

"Project Path/Genelists/AnalysisResults"

folder in text file format. Each gene list filename will begin with "timeseries_" and

end with the factor name. For example, the gene list for interaction based on

the p-value criterion will be "timeseries_p_interaction" and "timeseries_FDR_interaction"

for genes based on FDR criterion. The gene list for 'time' effect based on the p-value

criterion will be "timeseries_p_time" and "timeseries_FDR_time" for genes based on

FDR criterion. Users can use these gene lists for further analysis in ArrayTools.

**********************************

** References: **

**********************************

Benjamini, Y., and Hochberg, Y. (1995). Controlling the false discovery rate:

a practical and powerful approach to multiple testing. Journal of the Royal

Statistical Society Series B, 57, 289–300.
